# Endoscopic Treatment of Vesicoureteral Reflux in Children with Dextranomer/Hyaluronic Acid—A Single Surgeon's 6-Year Experience

**DOI:** 10.1155/2010/278012

**Published:** 2010-08-05

**Authors:** Hou-Chuan Chen, Chou-Ming Yeh, Chia-Man Chou

**Affiliations:** ^1^Division of Pediatric Surgery, Department of Surgery, Taichung Veterans General Hospital, 160, Sec 3, Chung-Kang Road, Taichung 40705, Taiwan; ^2^Department of Health, Taichung Hospital, Executive Yuan, 199, Sec 1, San Min Road, Taichung 40343, Taiwan

## Abstract

Endoscopic treatment for vesicoureteral reflux (VUR) has become an established alternative to long-term antibiotic prophylaxis and ureteral reimplantation. We present the outcome of endoscopic treatment with dextranomer/hyaluronic acid copolymer (Deflux) for VUR in children by a single surgeon at our institute from October 2003 to October 2009. We reviewed the cases of 150 patients (total 239 ureters), 56 girls (37%) and 94 boys (63%), with a mean age of 2.2 years and a median followup of 2.5 years (range 3–68 months). Among the 239 ureters treated, 67.4% (161/239) were cured with a single injection, and a second and third injection raised the cure rate to 86.6% (207/239) and 88.3% (211/239), respectively. None had postoperative ureteral obstruction.

## 1. Introduction

Vesicoureteric reflux (VUR) is a common problem encountered by pediatric urologists. Traditionally, if medical management with low-dose antibiotic prophylaxis failed, the only alternative was ureteral reimplant surgery [1]. Since Matouschek's initial description of the subureteric injection technique in 1981 [2] and the first clinical series reported by O'Donnell and Puri in 1984 [3], it has evolved into a therapeutic alternative to ureterocystostomy. Injectable agents, such as Teflon, bovine collagen, and Macroplastique, have all been used; however, concerns about efficacy and safety have limited their use [1, 4–6]. Since the first introduction into clinical use by Stenberg and Läckgren in 1995, dextranomer/hyaluronic acid copolymer (Deflux) has become an established alternative treatment for VUR in children [1]. We present the recent results of endoscopic treatment using the subtrigonal injection of Deflux for VUR in children by the same operator at our institute. 

## 2. Materials and Methods

We retrospectively reviewed all cases of subtrigonal injection performed with Deflux from October 2003 to October 2009 at Taichung Veteran General Hospital. All patients who entered into the study had vesicoureteric reflux, as determined by voiding cystourethrogram (VCUG). The radiological grading of VUR is according to the international system introduced by the International Reflux Study Committee in 1985 [7]. A total of 150 children, 1 month to 10 years old (mean age 2.2 years), received subtrigonal injection of Deflux for VUR. A subureteral or intraureteral injection, at the 6 o'clock position, delivered the material to correct VUR. Indications for intervention included breakthrough urinary tract infection, progressive renal scarring, poor compliance with medical therapy, nonresolution of VUR (>2 years), and parental preference [1, 2, 8]. Patients with more complicated urinary anomalies, such as ectopic ureter and neurogenic bladder, were also included. All procedures were carried out as day surgery, with the patients under general anesthetic. The procedures were all performed by the same surgeon. The technique comprises a hydrodistention-implantation technique with subureteric or intraureteric transurethral injection of Deflux with a Fr. 9.5 pediatric cystoscope, which is the same as the technique described in the literatures until we obtained a “bulge” with an elevated, inverted crescent shape of the orifice [4, 16]. Patients were maintained on their antibiotic prophylaxis until reflux was documented to be absent on postoperative VCUG or radionuclide cystogram at 3 months after injection. Patients were observed with dimercapto-succinic acid (DMSA) renal scan at the interval of 12 weeks postoperatively after surgery. Patients who failed initial injection were offered continued observation, a second injection, or ureteroneocystostomy. The outcomes were analyzed overall, and a subgroup analysis was carried out for patients with high grade VUR (grade IV to V).

## 3. Results

One hundred and fifty patients, 56 girls (37%) and 94 boys (63%), with a mean age of 2.2 years (range 1 mo–10 yr) underwent subtrigonal injection with Deflux from October 2003 to October 2009. Median followup duration was 2.5 years and ranged from 3 to 68 months. Eighty patients (53%) had bilateral VUR, 61 (41%) had unilateral VUR, and 9 (6%) had new contralateral VUR for a total of 239 ureters treated with a median followup of 2.5 years (range 2–62 mo). There are 9 (3.8%) ureters graded as I, 44 (18.4%) as II, 113 (47.3%) as III, 57 (23.8%) as IV, and 16 (6.7%) as V. The VCUG or radionuclide cystogram followup showed that the overall cure rate was 88.3% (211/239). The detail of the patients' data is shown in Table 1. Among the 239 ureters, 67.4% (161/239) were cured with a single injection. A second and third injection raised the cure rate to 86.6% (207/239) and 88.3% (211/239), respectively. Cure rate for grade I was 100% (9/9), grade II 91% (40/44), grade III 92% (104/113), grade IV 86% (49/57), and grade V 64% (10/16). One patient with unilateral grade IV VUR had 4 injections and then was cured. There were 143 children (229 ureters) with primary VUR, 2 children (3 ureters) with VUR secondary to neurogenic bladder, 2 children (3 ureters) with VUR secondary to ectopic kidneys, 2 children (2 ureters) with complex VUR, and one child (2 ureters) with VUR due to failed open ureteroneocystostomy. No ureter had postoperative ureteral obstruction. One had postoperative febrile UTI and subsided after intravenous antibiotic treatment. One had postoperative urinary retention and subsided after oral analgesic agents.

There were 73 ureters with high grade VUR, including 57 with grade IV VUR and 16 with grade V VUR. Overall cure rate was 80.8% (59/73), 5 (6.9%) ureters were downgraded to grade I, and 9 (12.3%) converted to open ureteral reimplantation. Complete resolution of vesicoureteral reflux after a single injection occurred in 33 ureters (45%), and 26 (36%) required more than 1 injection to correct VUR. No patients had postoperative vesicoureteral obstruction, gross hematuria, and febrile UTI.

## 4. Discussion

Our series demonstrates an overall patient cure rate of 88.3% (211/239) of the ureters among those who were cured needed more than a single injection. In addition, approximate 5% (12/239) was downgraded to grade I, and no further treatment was needed. These results are acceptable when we compare the cure rate obtained by other series shown in Table 2 or by open surgery, especially if we take into account the noninvasive nature of the technique [1, 5, 16]. Endoscopic treatment of high grade VUR has been evaluated in some series, and success rates vary from 55% to 90% [1]. In general, cure rates for high grade VUR are lower than that for low to moderate VUR whereas endoscopic treatment with Deflux has become an effective alternative to open ureteral reimplantation for high grade VUR [1]. The results of different series are shown in Table 3.

The complication rate in this series was low. Contralateral low-grade de novo VUR was present in 6% (9/239) of the patients treated for unilateral VUR—an incidence rate comparable to a previous report [1]. Conversion to open ureteral reimplantation was observed in 12 patients (8%), but the most common indications in our patients are due to parental decision and parental economic factor instead of a truly failed treatment. Ureteral obstruction has been described as a nonfrequent and temporary complication, and clinically none was observed in our series. Hematuria was absent or mild in most children and was limited to the first day postinjection [8]. The limitations of our study include that the data collected in the charts may be biased. 

## 5. Conclusion

Endoscopic correction is a safe, effective, and minimally invasive outpatient procedure for VUR in children. It demonstrated a cure rate of approximately 92% (138/150) of patients and 88% (211/239) of the ureters by using the bulking agent, Deflux by an experienced surgeon. Even high grade VUR, complex VUR, and failed open ureteroneocystostomy do not seem to adversely affect results.

## Figures and Tables

**Table 1:** The treatment results of various VUR grading in our patients.

Grade of VUR	No. of ureter	Complete resolution after				Downgrade to Gr. I	Injected volume (mean)	Conversion to open surgery
		1 inj.	2 inj.	3 inj.	4 Inj.			
I	9	9 (100%)	0	0	0	—	0.1–1 mL (0.6)	0
II	44	33 (75%)	7 (16%)	0	0	3 (7%)	0.2–1.3 mL (0.6)	1 (2%)
III	113	86 (76%)	18 (16%)	0	0	4 (4%)	0.1–1.7 mL (0.7)	5 (4%)
IV	57	31 (54%)	15 (26%)	2 (4%)	1 (2%)	2 (4%)	0.1–1.7 mL (0.9)	6 (10%)
V	16	2 (13%)	6 (38%)	2 (13%)	0	3 (18%)	0.3–2.3 mL (1.0)	3 (18%)

**Table 2:** Endoscopic treatment with Deflux for primary VUR in different series.

Series	Ureters	Injected volume	Followup	Success rate
Puri et al. [9]	1101	0.2–1.5 mL	3–46 mo	96%
Kirsch et al. [10]	139	0.8–2 mL	3–18 mo	93%
Yu and Roth [11]	162	1 mL	2–26 mo	93%
Pinto et al. [12]	86		3 mo	84%
Chen (2010)	239	0.1–2.3 mL	3–68 mo	88%

**Table 3:** Endoscopic treatment with Deflux for high grade VUR in different series.

Series	Ureters	Success rate		Downgrade to Gr. I	Conversion to op
		one inj.	> one inj.		
Kirsch et al. [5]	119	71–90%			
Dawrant et al. [13]	642	73%	16%	9%	2%
Menezes and Puri [14]	467	80%	16%	4%	0%
Altug et al. [15]	133	55%	19%		
Chen (2010)	73	45%	36%	7%	12%
